# Explainable Machine Learning Analysis of Perioperative Factors Associated with Clinically Significant Emergence Agitation After Pediatric Ophthalmic Surgery

**DOI:** 10.3390/medicina62061189

**Published:** 2026-06-19

**Authors:** Jung A Lim, Jonghae Kim, Minju Kong, Sang-Gyu Kwak

**Affiliations:** 1 Department of Anesthesiology and Pain Medicine, School of Medicine, Kyungpook National University, Daegu 41944, Republic of Korea; definitebud@naver.com; 2 Department of Anesthesiology and Pain Medicine, School of Medicine, Daegu Catholic University, Daegu 42472, Republic of Korea; usmed@cu.ac.kr (J.K.); minju2543@gmail.com (M.K.); 3 Department of Medical Statistics, School of Medicine, Daegu Catholic University, Daegu 42472, Republic of Korea

**Keywords:** child, delirium, emergence agitation, machine learning, ophthalmic surgery, sevoflurane

## Abstract

*Background and Objectives:* Emergence agitation (EA) is a common neurobehavioral disturbance during recovery from sevoflurane anesthesia in pediatric patients, particularly after ophthalmic surgery. Clinically deployable and rigorously validated risk stratification approaches remain limited. We aimed to develop and internally validate an explainable machine learning model to estimate individualized EA risk after pediatric ophthalmic surgery. *Materials and Methods:* This retrospective cohort study included 1029 children aged 3–7 years who underwent ophthalmic surgery under sevoflurane anesthesia between 2016 and 2025. EA was defined as clinically significant agitation requiring active management in the post-anesthesia care unit. Four machine learning algorithms (regularized logistic regression, random forest, XGBoost, and CatBoost) were developed using stratified patient-level 5-fold cross-validation. Performance was evaluated using pooled out-of-fold predictions. Discrimination, calibration, and classification metrics at the optimal Youden threshold were assessed. SHAP analysis was applied for interpretability. *Results:* EA occurred in 543 patients (52.8%). XGBoost showed comparable discrimination with slightly higher AUPRC (0.827) and sensitivity (0.796) compared with other models, while maintaining acceptable specificity (0.728). Calibration demonstrated good agreement between predicted and observed risk. SHAP identified airway management and anesthetic-related variables as key contributors. *Conclusions:* ML-based analysis identified clinically relevant perioperative factors associated with emergence agitation and may provide preliminary insight into perioperative risk stratification pending external validation. External validation is required before clinical implementation.

## 1. Introduction

Emergence agitation (EA) is a transient but clinically significant neurobehavioral disturbance observed during early recovery from general anesthesia in pediatric patients. It is characterized by restlessness, inconsolability, crying, disorientation, and purposeless movements, and is particularly common following sevoflurane anesthesia [1,2,3]. The reported incidence of EA varies widely, ranging from 10% to 80%, depending on patient age, anesthetic exposure, surgical type, and diagnostic criteria [4,5,6]. Ophthalmic procedures, including strabismus surgery, have consistently been identified as high-risk settings for EA [7,8]. Although often self-limiting, EA can lead to surgical site disruption, accidental removal of medical devices, increased sedative or opioid administration, prolonged PACU stay, and significant caregiver distress [5,9,10]. Therefore, early identification of children at elevated risk of EA is of considerable clinical importance.

Numerous perioperative factors have been associated with EA development. Volatile anesthetic exposure and depth [2,8]; airway management techniques, such as endotracheal intubation versus laryngeal mask airway (LMA Flexible™ Single Use; Teleflex Medical, Dublin, Ireland) [11,12]; pharmacologic prophylaxis strategies [13,14]; and induction agents, including ketamine or midazolam [15,16], have all been implicated. Risk scale development studies have attempted to combine these variables into structured prediction tools [17]. However, several important limitations persist in the existing literature.

First, many published studies focus on hypothesis-driven association testing rather than clinically deployable risk prediction. Regression-derived odds ratios provide population-level inference but do not directly translate into individualized probability estimates needed for bedside decision-making. Second, conventional multivariable regression models assume linearity and additive effects among predictors. In anesthetic practice, perioperative variables are often interdependent; for example, airway device selection influences neuromuscular blockade use, airway stimulation, opioid requirements, and extubation timing. When such correlated predictors are analyzed within linear models, multicollinearity may distort effect estimates and compromise interpretability. Third, many prior investigations are limited by heterogeneous surgical populations or relatively small sample sizes, which may reduce model generalizability and limit procedural specificity [12,13].

Another critical methodological concern is insufficient validation rigor. Several EA studies report predictive associations without robust internal validation or strategies to prevent optimistic bias. Standard cross-validation approaches may inadvertently introduce data leakage when repeated procedures from the same patient are distributed across folds, thereby inflating apparent performance. Contemporary prediction research increasingly emphasizes methodological transparency, reproducibility, and leakage prevention, as reflected in emerging reporting guidance such as TRIPOD-Code [18]. Without such rigor, predictive performance estimates may not be reliable for clinical deployment.

Machine learning (ML) methods offer a flexible framework to address these limitations. Unlike traditional regression models, ML algorithms can accommodate nonlinear relationships and complex interactions without prespecified parametric assumptions. However, the clinical adoption of ML requires not only predictive accuracy but also interpretability. Black-box models lacking transparency may undermine clinician trust and hinder implementation. Shapley additive explanations (SHAP) provide a theoretically grounded method to quantify feature contributions at both global and individual levels, thereby enhancing interpretability and enabling clinically meaningful explanation of model outputs [19].

Pediatric ophthalmic surgery represents a clinically relevant setting for developing a perioperative prediction model because of the relatively high incidence of emergence agitation and broadly shared perioperative management characteristics [7,8]. An internally validated model capable of generating individualized perioperative risk stratification during anesthetic management could inform perioperative planning, including the selection of airway devices, anesthetic strategies, and postoperative monitoring. Such risk-stratified approaches may help inform perioperative decision-making and may improve patient safety while avoiding unnecessary interventions in low-risk patients.

Because standardized agitation or delirium scales (e.g., the Pediatric Anesthesia Emergence Delirium [PAED] scale) are not routinely documented in retrospective perioperative datasets, clinically documented agitation requiring active PACU management represents a pragmatic and clinically meaningful endpoint for prediction modeling.

Compared with previous emergence agitation studies, the present study incorporates several methodological features intended to improve robustness, interpretability, and reproducibility. First, we focused on a pediatric ophthalmic surgery cohort with a relatively high incidence of emergence agitation and broadly shared perioperative characteristics. Second, we implemented patient-level grouped cross-validation with pooled out-of-fold (OOF) evaluation to minimize information leakage and optimistic bias arising from repeated procedures in the same patient. Third, we incorporated SHAP-based explainability analysis to improve transparency and clinical interpretability of machine learning predictions. Finally, the complete analysis pipeline was made publicly available to enhance reproducibility and methodological transparency.

## 2. Methods

### 2.1. Study Design and Population

This retrospective cohort study was conducted at Daegu Catholic University Medical Center and approved by the Institutional Review Board of Daegu Catholic University Medical Center (DCUMC 2026-01-052). The requirement for written informed consent was waived because of the retrospective design and the use of de-identified data. The predefined study period ended in December 2025 because the study protocol was prepared in January 2026 using retrospectively available data up to that time point.

We reviewed the electronic medical records of pediatric patients who underwent ophthalmic surgery under general anesthesia between January 2016 and December 2025. Eligible patients were aged 3–7 years and had American Society of Anesthesiologists (ASA) physical status I or II. All procedures were performed under sevoflurane maintenance anesthesia. Patients were excluded if they had ASA physical status ≥ III, received maintenance anesthesia other than sevoflurane, underwent combined or non-eligible surgical procedures, received anesthetic or analgesic agents outside the predefined institutional protocol, or had insufficient medical records.

After applying these criteria, 1029 patients were included in the final analysis. If multiple procedures were performed in the same patient during the study period, each procedure was included as an observation; however, patient-level grouping was strictly maintained during model validation to prevent data leakage. Among the 1029 included procedures, data were derived from 1002 unique patients. A total of 25 patients underwent more than one eligible procedure during the study period, accounting for 52 procedures overall. The ophthalmic surgery cohort included both eyelid procedures (e.g., Hotz–Celsus operation) and extraocular muscle surgeries performed under standardized pediatric sevoflurane anesthesia protocols.

### 2.2. Outcome Definition

The primary outcome was EA, defined as the presence of hyperactive behavioral disturbances during early recovery in the post-anesthesia care unit (PACU). EA was characterized by restlessness, inconsolability, or disorientation requiring active clinical management in the PACU, such as pharmacologic intervention or supportive behavioral management [1,3,5,6]. Because this study was retrospective, raw itemized Pediatric Anesthesia Emergence Delirium (PAED) scale scores were not consistently archived as structured quantitative variables in the electronic medical record system throughout the study period. However, our institution maintained a standardized PACU management protocol during the study period in which attending anesthesiologists routinely assessed emergence agitation in real time using the Pediatric Anesthesia Emergence Delirium (PAED) scale framework and contemporaneous behavioral assessment principles described in prior pediatric emergence delirium studies [20,21]. In our institutional practice, clinically significant emergence agitation requiring active management prompted standardized rescue treatment, including fentanyl administration. Therefore, outcome labeling in this retrospective cohort was based primarily on contemporaneous behavioral documentation and clinician-recorded EA assessment under the standardized institutional protocol rather than being defined solely by pharmacologic intervention. Pharmacologic intervention, including fentanyl administration, was considered supportive clinical context but was not independently sufficient for outcome labeling.

Hypoactive features, such as reduced responsiveness or decreased interaction, were not considered consistent with EA.

Outcome labeling was determined retrospectively from routine PACU documentation and medication records. To prevent data leakage, only variables available before PACU arrival were included as predictors in model development. This definition reflects clinically actionable agitation requiring intervention and is clinically relevant because emergence delirium has been associated with subsequent postoperative behavioral disturbances in pediatric patients [22].

### 2.3. Candidate Predictors

Candidate predictors were selected based on clinical plausibility and prior literature [7,8,11,12,13,14,15,16,17], and availability before the occurrence of EA. Only variables routinely documented in the electronic medical record and available before PACU arrival were considered, to ensure temporal validity and prevent information leakage.

We focused on perioperative variables that are clinically accessible and relevant to anesthetic management, including airway strategy, induction regimen, neuromuscular blockade dose, and intraoperative opioid administration. During the study period, no institutional protocol mandated airway device selection for pediatric ophthalmic surgery. Therefore, the choice between endotracheal intubation and laryngeal mask airway was made at the discretion of the attending anesthesiologist according to individual clinical judgment and practice preference.

Continuous variables were retained in their original scale without categorization to preserve predictive information and avoid arbitrary thresholding. Categorical variables were encoded according to algorithm-specific requirements. Variables that were not consistently recorded (e.g., standardized anxiety scores or intraoperative depth-of-anesthesia monitoring indices) were not included to maintain data completeness and model robustness. Some predictors were clinically interdependent; however, all were retained because the aim was prediction rather than causal effect estimation. Variables included in model development were routinely recorded perioperative variables with minimal missingness. Cases with insufficient or substantially incomplete records were excluded during cohort selection. Because no substantial missingness was present among the included candidate predictors, complete-case analysis was performed and no imputation procedure was required.

### 2.4. Model Development and Validation

Four supervised machine learning algorithms were developed and compared: regularized logistic regression with L2 penalty, random forest, extreme gradient boosting (XGBoost), and CatBoost. All models were trained to predict the probability of EA as a binary outcome. To obtain an unbiased estimate of model performance and to prevent information leakage, we employed stratified group 5-fold cross-validation at the patient level. Stratification ensured that the proportion of EA cases was approximately balanced across folds, while grouping by unique patient identifier ensured that all procedures from the same patient were assigned to the same fold. This approach prevented the same patient from appearing in both training and validation data, thereby minimizing optimistic bias. Within each cross-validation iteration, four folds were used for model training and hyperparameter tuning, and the remaining fold was reserved exclusively for validation. Hyperparameter tuning was conducted using cross-validation restricted to the training portion of each fold, and the held-out validation fold was not used at any stage of model tuning.

For logistic regression, grid search was performed over the inverse regularization strength (C), which controls the degree of L2 regularization, with smaller values corresponding to stronger penalization. For tree-based models (random forest, XGBoost, and CatBoost), randomized search was applied across predefined hyperparameter ranges. The complete hyperparameter search space for each algorithm is provided in Supplementary Table S1. Nested cross-validation was not implemented, and hyperparameter optimization was performed within the same cross-validation framework used for model evaluation. The optimal hyperparameters were selected based on the highest mean area under the receiver operating characteristic curve (AUROC) within the training folds. After tuning, each model with the selected hyperparameters was refit on the entire training portion of the fold and evaluated on the held-out validation fold. This process was repeated across all five folds. Predicted probabilities from each validation fold were pooled to generate OOF predictions for the entire dataset. Because each OOF prediction was generated from a model that had not been trained on that specific observation, these pooled predictions provided an unbiased estimate of model performance.

Model comparison considered cross-validated discrimination primarily on the basis of AUROC, with AUPRC and fold-wise stability used as complementary criteria. The model demonstrating the most favorable overall balance of discrimination metrics and stability across folds was selected as the primary model for further calibration and explainability analyses. This validation framework was adopted to reduce the risk of overfitting and to provide robust and reproducible estimates of model performance in accordance with contemporary recommendations for prediction modeling studies.

### 2.5. Model Performance Evaluation

Model performance was evaluated using pooled OOF predicted probabilities generated from stratified group 5-fold cross-validation. Because each OOF prediction was obtained from a model that had not been trained on the corresponding observation, these pooled predictions provided an unbiased estimate of model performance. Discrimination was assessed using the AUROC, which reflects the model’s ability to distinguish between patients with and without EA across all possible probability thresholds. In addition, the AUPRC was calculated to provide complementary information regarding performance under the observed class distribution. Although the incidence of EA was relatively balanced, AUPRC was included because it is more sensitive to changes in positive class prediction performance and may offer additional insight into model behavior.

Calibration was evaluated to determine the agreement between predicted probabilities and observed event rates. Calibration plots were constructed by grouping predicted probabilities into deciles and comparing the mean predicted probability with the observed EA rate within each decile. Visual comparison with the line of perfect calibration was performed to assess systematic over- or underestimation of risk.

For classification-based performance assessment, an optimal probability threshold for each model was determined using the Youden index (maximum sensitivity + specificity − 1) derived from OOF predictions. The Youden index was used as a pragmatic approach for balanced classification performance rather than reflecting a clinically optimized decision threshold. At this threshold, sensitivity, specificity, positive predictive value (PPV), negative predictive value (NPV), overall accuracy, and F1-score were calculated. These metrics were reported to facilitate clinical interpretability and to illustrate the trade-off between false-positive and false-negative predictions. All performance metrics were derived exclusively from OOF predictions to maintain independence between training and evaluation data and to minimize the risk of optimistic bias.

### 2.6. Explainability Analysis 

To enhance interpretability and facilitate clinical translation, SHAP analysis was applied to the XGBoost model, which demonstrated slightly higher AUPRC and sensitivity among the evaluated models [19]. SHAP is a game-theoretic approach that decomposes each individual prediction into additive contributions of the input features, thereby quantifying how each predictor influences the model output relative to the baseline risk. Global interpretability was assessed by calculating the mean absolute SHAP value for each feature across all patients, which reflects the overall magnitude of contribution to model predictions. Features with larger mean absolute SHAP values were considered more influential in determining predicted risk. A SHAP summary plot was generated to simultaneously display feature importance, directionality of effect, and distribution of feature values. In this plot, each dot represents an individual patient, with color indicating the relative magnitude of the feature value and horizontal position representing the SHAP value (impact on the log-odds of EA).

Local interpretability was evaluated by examining SHAP values at the individual level, allowing identification of which predictors increased or decreased predicted EA risk for specific patients. This approach enables clinically meaningful interpretation of high-risk predictions and supports individualized perioperative risk assessment. All SHAP analyses were conducted using pooled OOF predictions to ensure that feature attribution was based on models that had not been trained on the corresponding observations. This strategy minimized optimistic bias and improved the robustness of explainability results. SHAP value was expressed on the log-odds scale, consistent with the model output. Positive SHAP values indicate an increase in predicted EA risk relative to the baseline, whereas negative values indicate a decrease in predicted risk.

### 2.7. Statistical Analysis

Continuous variables are presented as mean ± standard deviation, and categorical variables as number (percentage). Baseline characteristics were summarized for the overall cohort and stratified according to EA status. All machine learning analyses were conducted using Python (version 3.12.12) in the Google Colaboratory (Google LLC, Mountain View, CA, USA) cloud-based environment. The following libraries were used: scikit-learn (version 1.6.1), XGBoost (version 3.2.0), CatBoost (version 1.2.10), and SHAP (version 0.50.0). Random seeds were fixed across all algorithms to ensure reproducibility of model training and validation procedures. Descriptive statistical analyses and baseline group comparisons were primarily conducted using IBM SPSS Statistics (version 24.0; IBM Corp., Armonk, NY, USA), whereas additional statistical visualization and supplementary analyses were performed using R (version 4.5.1; R Foundation for Statistical Computing, Vienna, Austria). For descriptive comparisons between the EA and no-EA groups, continuous variables were analyzed using two sample t-test, and categorical variables were compared using chi-square test. Formal a priori sample size calculation was not performed because of the retrospective study design. However, the dataset included 543 EA events and a sufficient events-per-variable ratio according to conventional recommendations for prediction model development.

Model performance metrics were derived exclusively from pooled OOF predictions generated through stratified group 5-fold cross-validation, as described above, to minimize optimistic bias and enhance robustness of performance estimates. The complete reproducible analysis pipeline, including preprocessing, stratified group cross-validation, pooled OOF performance estimation, and SHAP-based explainability analysis, is available at: https://github.com/sanggyu3939/EA-ML-ophthalmic-surgery (accessed on 17 June 2026). The dataset is not publicly available because of patient confidentiality and institutional regulations.

## 3. Results

### 3.1. Study Population

**Figure 1:**
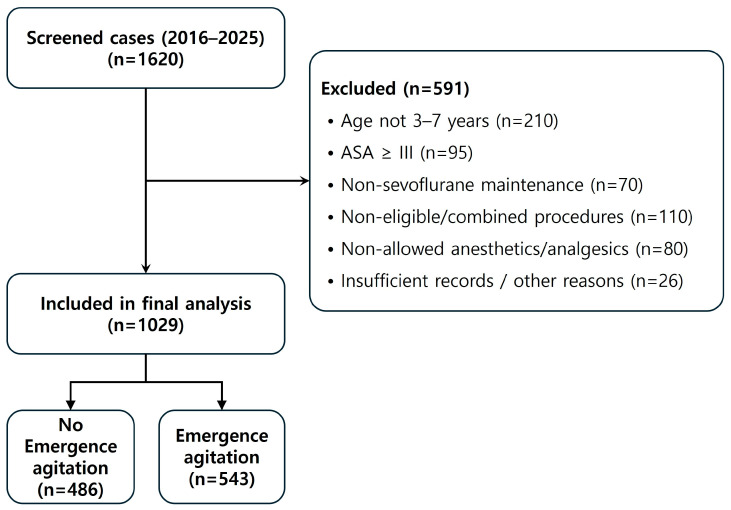
Study flow diagram of patient selection. Emergence agitation (EA) was defined as hyperactive behavioral disturbance requiring clinical management in the post-anesthesia care unit (PACU), including restlessness, inconsolability, or disorientation.

Between 2016 and 2025, a total of 1620 pediatric cases undergoing ophthalmic surgery were screened for eligibility (Figure 1). A total of 591 cases were excluded for the following reasons: age outside 3–7 years (*n* = 210), ASA physical status ≥ III (*n* = 95), non-sevoflurane maintenance (*n* = 70), non-eligible or combined procedures (*n* = 110), use of non-allowed anesthetics/analgesics (*n* = 80), or insufficient records/other reasons (*n* = 26). The final analytic cohort therefore comprised 1029 patients. EA occurred in 543 patients (52.8%), whereas 486 patients (47.2%) did not experience EA.

### 3.2. Baseline and Perioperative Characteristics

Baseline, surgical, and anesthetic characteristics are summarized in Table 1. The mean age was 5.38 ± 1.15 years, and the mean body mass index (BMI) was 16.86 ± 2.69 kg/m^2^. Most patients were classified as ASA physical status I (97.6%), and recent upper respiratory infection (URI) symptoms were documented in 5.1%. Marked between-group differences were observed in airway management and induction regimen. Endotracheal tube use was more common in the EA group than in the no-EA group (74.6% vs. 24.5%, *p* < 0.001). Regarding induction regimen, ketamine alone was the most common approach overall (62.0%) and was more prevalent in the EA group than in the no-EA group (77.2% vs. 45.1%, *p* < 0.001). In contrast, combined ketamine–midazolam induction was less frequent in the EA group (8.5% vs. 33.3%, *p* < 0.001).

Perioperative medication and time variables also differed by EA status. Patients with EA received higher rocuronium doses (0.72 ± 0.26 vs. 0.54 ± 0.25 mg/kg) and had longer anesthesia and operation times than those without EA (anesthesia time: 72.36 ± 22.31 vs. 66.07 ± 19.90 min; operation time: 38.16 ± 20.87 vs. 31.85 ± 17.41 min). Intraoperative fentanyl dose was comparable between groups.

### 3.3. Predictive Performance of Machine Learning Models

The predictive performance of the machine learning models is summarized in Table 2. Model discrimination was evaluated using stratified group 5-fold cross-validation, and performance metrics were derived from pooled OOF predictions. Across cross-validation folds, logistic regression and XGBoost achieved similar mean AUROC values (both 0.809), followed by random forest (0.801) and CatBoost (0.790). In terms of precision–recall performance, XGBoost showed slightly higher mean AUPRC (0.827 ± 0.024), whereas logistic regression and random forest demonstrated comparable but lower AUPRC values (both approximately 0.806). CatBoost showed modestly lower performance in both AUROC and AUPRC. Consistent patterns were observed in pooled OOF evaluation. XGBoost achieved an AUROC of 0.810 and an AUPRC of 0.827, while logistic regression showed a comparable AUROC (0.807) but lower AUPRC (0.799). Random forest and CatBoost exhibited slightly lower performance across both discrimination metrics. Overall, differences in AUROC between models were modest, whereas XGBoost showed slightly higher AUPRC, suggesting improved identification of higher-risk patients. Pairwise DeLong tests using pooled OOF predictions demonstrated no statistically significant AUROC differences between XGBoost and the other evaluated models (all *p* > 0.05). ROC and precision–recall curves for logistic regression, random forest, and CatBoost are provided in Supplementary Figures S1–S3 for comparison.

### 3.4. Classification Performance at the Optimal Youden Threshold

Classification performance at the optimal Youden threshold derived from pooled OOF predictions is presented in Table 3. For each model, the decision threshold was determined by maximizing the Youden index (sensitivity + specificity − 1) on the OOF ROC curve. Among the evaluated models, XGBoost showed higher sensitivity (0.796) while maintaining acceptable specificity (0.728), resulting in a PPV of 0.766, NPV of 0.761, overall accuracy of 0.764, and the highest F1-score (0.780). Logistic regression showed higher specificity (0.805) but lower sensitivity (0.727), indicating a more conservative classification pattern. Random forest exhibited intermediate performance, whereas CatBoost demonstrated relatively high specificity (0.796) but lower sensitivity (0.680). Overall, XGBoost showed a favorable balance between sensitivity and specificity at the optimal threshold, whereas logistic regression favored higher specificity at the expense of reduced sensitivity.

### 3.5. Model Discrimination and Calibration

**Figure 2:**
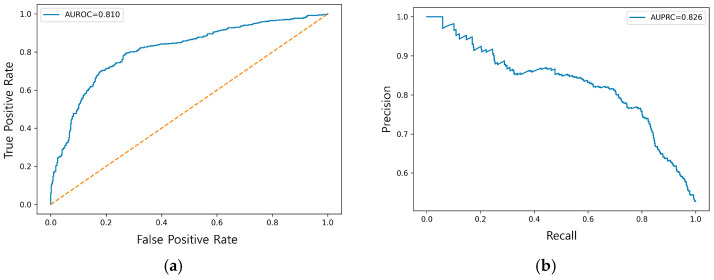
Receiver operating characteristic (ROC) and precision–recall (PR) curves for the XGBoost model based on out-of-fold (OOF) predictions. ROC curve (**a**) and PR curve (**b**) for the XGBoost model using pooled OOF predicted probabilities obtained from stratified group 5-fold cross-validation. The area under the ROC curve (AUROC) and area under the precision–recall curve (AUPRC) are shown. All predictions were generated at the patient level to prevent data leakage from repeated procedures. The dashed diagonal line represents the reference line corresponding to random classification performance.

Model discrimination based on pooled OOF predictions is illustrated in Figure 2. The ROC curves demonstrated good overall separation between patients with and without EA across all evaluated algorithms. XGBoost and logistic regression showed comparable ROC performance, with curves consistently above those of random forest and CatBoost across most false-positive rate ranges. Precision–recall curves similarly indicated improved performance of XGBoost in identifying high-risk patients, reflected by higher precision at comparable recall levels.

**Figure 3:**
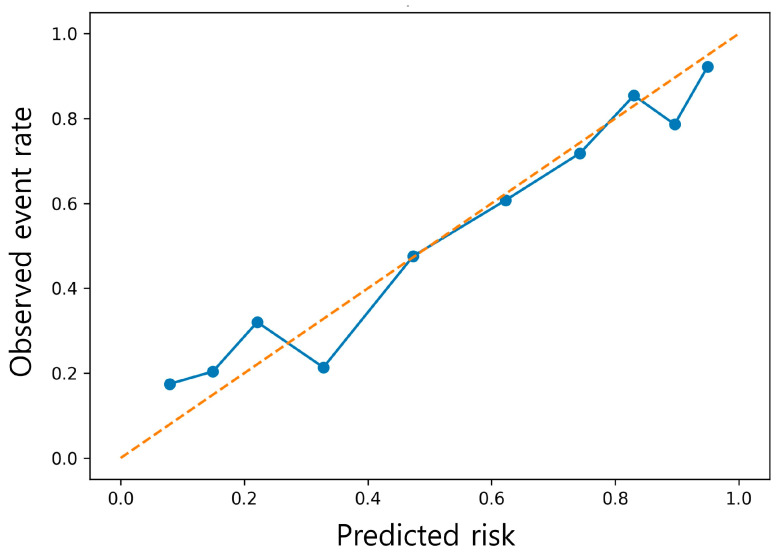
Calibration curve for the XGBoost model based on out-of-fold predictions. Observed event rates are plotted against predicted probabilities using pooled out-of-fold predictions from stratified group 5-fold cross-validation. Predicted probabilities were grouped into deciles. The dashed diagonal line represents perfect calibration. The orange dashed diagonal line represents perfect calibration.

Calibration performance is presented in Figure 3. Across deciles of predicted risk, observed event rates were generally aligned with predicted probabilities for all models. XGBoost demonstrated slightly improved agreement between predicted and observed risk in the intermediate-to-high risk range, whereas logistic regression exhibited mild underestimation in the highest decile. Calibration plots showed that predicted probabilities were well aligned with observed event rates across the risk spectrum.

### 3.6. SHAP-Based Explainability Results

**Figure 4:**
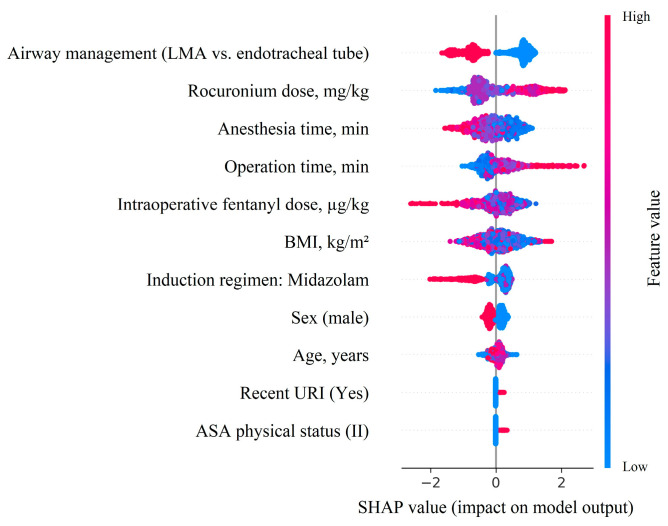
SHAP summary plot for the XGBoost model. Each dot represents an individual patient. Red indicates higher feature values and blue indicates lower feature values. SHAP value represents the impact of each feature on the model output (log-odds of emergence agitation). Airway management was coded with LMA as the displayed feature category; thus, lower SHAP value indicates lower predicted EA risk with LMA relative to endotracheal tube use. ASA, American Society of Anesthesiologists; BMI, body mass index; LMA, laryngeal mask airway; SHAP, Shapley additive explanations; URI, upper respiratory infection.

To enhance interpretability and facilitate clinical translation, SHAP analysis was applied to the best-performing model, XGBoost, using pooled OOF predictions (Figure 4). The SHAP summary plot illustrates both the magnitude and direction of each predictor’s contribution to the model output at the individual patient level.

Airway management was identified as the most influential feature in predicting EA. Endotracheal tube use was associated with higher predicted EA risk in the model, whereas LMA use contributed to lower predicted risk. Rocuronium dose was also an important contributor to model output; however, this finding should be interpreted in the context of its close relationship with airway management strategy. Induction regimen also showed substantial influence, with ketamine-only induction contributing to higher predicted risk, whereas combined ketamine–midazolam induction contributed to lower predicted risk. Intraoperative fentanyl dose, anesthesia duration, and operation time exhibited moderate contributions, with higher values generally associated with increased predicted risk. In contrast, demographic variables such as age and sex showed relatively smaller SHAP magnitudes, suggesting that perioperative airway and anesthetic management variables played a more dominant role in model-derived EA risk estimation than baseline patient characteristics.

A comparison of global feature importance across all evaluated algorithms is presented in Supplementary Table S2. Notably, airway management consistently ranked as the most influential feature across models, supporting the robustness of this finding beyond a single algorithm. Overall, the explainability analysis demonstrated that the XGBoost model identified clinically plausible patterns in how perioperative variables contributed to predicted EA risk.

## 4. Discussion

In this retrospective cohort study of pediatric patients undergoing ophthalmic surgery, we performed an explainable machine learning analysis to identify perioperative factors associated with EA. Among the evaluated algorithms, XGBoost showed comparable performance across discrimination, classification metrics, and calibration. Importantly, model performance was estimated using stratified group 5-fold cross-validation with pooled OOF predictions, minimizing optimistic bias and enhancing methodological rigor.

Although logistic regression achieved comparable AUROC values, XGBoost showed slightly higher AUPRC and sensitivity, suggesting improved identification of higher-risk patients. These findings suggest that the predictive structure within this relatively homogeneous perioperative dataset may be captured reasonably well by both conventional regression and nonlinear machine learning approaches. Therefore, the primary advantage of XGBoost may relate more to improved precision–recall performance and modeling of complex feature interactions rather than substantially superior overall discrimination.

The explainability analysis provided clinically meaningful insights. Airway management emerged as the most influential feature, with endotracheal tube use associated with increased predicted EA risk and LMA use associated with reduced risk. This finding is consistent with prior randomized and observational studies reporting higher perioperative airway stimulation and respiratory adverse events with endotracheal intubation compared with LMA [11,12]. Increased airway irritation may contribute to heightened sympathetic activation during emergence, potentially facilitating agitation. Rocuronium dose also contributed to model predictions. Because neuromuscular blockade is routinely administered during tracheal intubation, rocuronium exposure in this dataset likely reflects differences in airway management strategy rather than an independent causal effect on emergence agitation. The higher predicted risk associated with ketamine-only induction may reflect its dissociative properties and potential to increase perceptual disturbance during emergence. In contrast, the lower predicted risk observed with combined ketamine–midazolam induction may reflect the anxiolytic and sedative effects of benzodiazepines, which could mitigate agitation during recovery.

Interestingly, demographic variables such as age and sex exhibited relatively smaller contributions in SHAP analysis compared with perioperative management variables. While younger age has been reported as a risk factor in broader pediatric populations [1,6], our pediatric ophthalmic surgery cohort of children aged 3–7 years may have attenuated age-related variability. This observation underscores the value of developing surgery-specific prediction models rather than relying on generalized pediatric EA risk factors.

The relatively high incidence of EA observed in this cohort (52.8%) is consistent with previous reports describing increased susceptibility among pediatric ophthalmic surgery populations [7,8]. Several factors likely contribute to this tendency, including the young age of the patients, the use of sevoflurane anesthesia, and perioperative sensory disturbances (e.g., blurred vision) associated with ophthalmic procedures. By focusing on a pediatric ophthalmic surgery cohort with relatively consistent perioperative management characteristics, we developed a clinically focused dataset that may enhance the stability and interpretability of the prediction model compared with analyses derived from highly heterogeneous surgical populations.

Previous attempts to develop EA risk scales have primarily relied on multivariable logistic regression frameworks [17]. Although these models provide interpretable effect estimates, they assume linearity and additive effects among predictors. In anesthetic practice, however, airway device choice, neuromuscular blockade dosing, anesthetic depth, and opioid administration are interdependent decisions. Machine learning approaches may better accommodate such complex interactions. Importantly, the present study incorporated strict patient-level grouping during cross-validation to prevent leakage from repeated procedures, a methodological safeguard that has not consistently been reported in prior EA prediction research.

From a clinical perspective, individualized EA risk estimation may inform perioperative planning. For patients predicted to be at higher risk, the model may support consideration of strategies such as airway device selection, optimization of induction regimen, or closer postoperative monitoring. Conversely, in lower-risk patients, it may help avoid overly aggressive prophylactic strategies. However, external validation and prospective impact studies are necessary before clinical implementation. Accordingly, the model should be interpreted as hypothesis-generating and decision-supportive rather than practice-changing at this stage.

This study has several strengths. First, we focused on a pediatric ophthalmic surgery population with a high and well-characterized incidence of EA. Second, we applied stratified group cross-validation to prevent optimistic bias and ensure independence between training and evaluation data. Third, we integrated SHAP-based explainability to enhance transparency and clinical interpretability. Finally, the full analysis pipeline is publicly available to support reproducibility.

Several limitations should be acknowledged. This was a single-center retrospective study, and practice patterns may limit generalizability to other institutions. Therefore, external validation in independent cohorts is required to confirm generalizability across different clinical settings. In addition, several perioperative management variables included in the model may partially reflect local anesthetic practice patterns or clinician preferences. Therefore, some predictive patterns identified by the model may represent local perioperative practice characteristics rather than universally generalizable causal relationships. Accordingly, variables such as airway management strategy should be interpreted primarily as predictive perioperative features within the observed institutional context rather than directly modifiable causal determinants. External validation across multiple institutions with different anesthetic practices will be important to evaluate model transportability and generalizability. Although the study focused on pediatric ophthalmic surgery, the included procedures comprised both eyelid and extraocular muscle surgeries, which may introduce procedural heterogeneity. Therefore, procedure-specific external validation will be necessary before applying the model to a specific ophthalmic surgical subgroup.

Although emergence agitation was routinely assessed using the institutional PAED-based PACU protocol, raw itemized PAED scores were not consistently archived as structured quantitative variables in the retrospective electronic medical records. Therefore, outcome ascertainment relied on clinically documented agitation requiring active management in the PACU, and some degree of misclassification is possible. Because postoperative pain and agitation may partially overlap during early PACU recovery, some degree of residual misclassification cannot be completely excluded in this retrospective cohort. However, this pragmatic definition reflects clinically meaningful events requiring intervention in routine practice.

Therefore, the model should be interpreted as predicting clinically actionable EA rather than strictly scale-defined agitation. Additionally, several potentially relevant perioperative factors, including preoperative anxiety, postoperative pain severity, depth-of-anesthesia monitoring indices, emergence time, and standardized pain assessment or analgesic administration criteria, were not consistently available for inclusion because of the retrospective design. Because several predictors became fully available only during intraoperative management, the present framework should be interpreted as a perioperative risk stratification model rather than a strictly preoperative prediction tool. These unmeasured factors may have influenced both perioperative management decisions and emergence agitation risk. Although airway management strategy showed strong association with predicted EA risk, the present observational study design does not permit causal inference regarding whether changing airway devices would directly alter EA risk. Because nested cross-validation was not performed, some degree of optimistic bias in performance estimation cannot be completely excluded. Although the events-per-variable ratio was adequate according to conventional regression modeling recommendations, larger multicenter datasets may be required to fully optimize and externally validate more complex machine learning architectures.

## 5. Conclusions

In this procedure-specific cohort of pediatric ophthalmic surgery, we developed an explainable machine learning framework for identifying perioperative factors associated with emergence agitation. Using stratified patient-level cross-validation and pooled out-of-fold evaluation, XGBoost demonstrated comparable discrimination, classification performance, and calibration relative to the other evaluated models. Explainability analysis identified airway management and anesthetic strategy as important contributors to model-derived EA risk within the observed institutional context. Although these findings may provide preliminary insight into perioperative risk stratification, the results should be considered hypothesis-generating pending external validation and prospective clinical evaluation.

## Figures and Tables

**Table 1:** Baseline, surgical, and anesthetic characteristics according to emergence agitation.

Variable	Overall (*n* = 1029)	No EA (*n* = 486)	EA (*n* = 543)	*p*-Value
Age, years	5.38 ± 1.15	5.37 ± 1.21	5.40 ± 1.09	0.723
BMI, kg/m^2^	16.86 ± 2.69	16.99 ± 2.60	16.74 ± 2.77	0.144
Sex				0.011
Male	494 (48.0)	213 (43.8)	281 (51.7)	
Female	535 (52.0)	273 (56.2)	262 (48.3)	
ASA physical status				0.374
I	1004 (97.6)	472 (97.1)	532 (98.0)	
II	25 (2.4)	14 (2.9)	11 (2.0)	
Recent URI				0.194
No	977 (94.9)	466 (95.9)	511 (94.1)	
Yes	52 (5.1)	20 (4.1)	32 (5.9)	
Rocuronium dose, mg/kg *	0.66 ± 0.27	0.54 ± 0.25	0.72 ± 0.26	<0.001
Intraoperative fentanyl dose, µg/kg	0.89 ± 0.30	0.89 ± 0.33	0.89 ± 0.27	0.881
Anesthesia time, min	69.39 ± 21.43	66.07 ± 19.90	72.36 ± 22.31	<0.001
Operation time, min	35.18 ± 19.56	31.85 ± 17.41	38.16 ± 20.87	<0.001
Surgical procedure				<0.001
Hotz–Celsus	582 (56.6)	292 (60.1)	290 (53.4)	
RLR	319 (31.0)	116 (23.9)	203 (37.4)	
BLR	108 (10.5)	68 (14.0)	40 (7.4)	
Others†	20 (1.9)	10 (2.0)	10 (1.8)	
Airway management				<0.001
Endotracheal tube	524 (50.9)	119 (24.5)	405 (74.6)	
LMA	505 (49.1)	367 (75.5)	138 (25.4)	
Induction regimen				<0.001
Ketamine	638 (62.0)	219 (45.1)	419 (77.2)	
Midazolam	183 (17.8)	105 (21.6)	78 (14.4)	
Ketamine + Midazolam	208 (20.2)	162 (33.3)	46 (8.5)	

Values are presented as frequency (percentage) or mean ± standard deviation. *p* values were calculated using two-sample *t*-test for continuous variables and chi-square test for categorical variables, as appropriate. * Calculated among patients who received rocuronium. † Others include procedures with frequency <1%. ASA, American Society of Anesthesiologists; BLR, bilateral lateral rectus recession; BMI, body mass index; EA, emergence agitation; LMA, laryngeal mask airway; RLR, recession of lateral rectus; URI, upper respiratory infection.

**Table 2:** Predictive performance of machine learning models for emergence agitation using 5-fold stratified group cross-validation.

Model	5-Fold Cross-Validation		Pooled Out-of-Fold Predictions	
	AUROC	AUPRC	AUROC	AUPRC
Logistic regression	0.809 ± 0.036	0.806 ± 0.058	0.807	0.799
XGBoost	0.809 ± 0.029	0.827 ± 0.024	0.810	0.827
Random forest	0.801 ± 0.033	0.806 ± 0.033	0.800	0.799
CatBoost	0.790 ± 0.029	0.804 ± 0.026	0.789	0.803

Performance was evaluated using 5-fold stratified group cross-validation at the patient level to prevent data leakage from repeated procedures in the same patient. Values under “5-fold cross-validation” are presented as mean ± standard deviation across the five validation folds. Values under “Pooled out-of-fold predictions” were derived from pooled out-of-fold predicted probabilities across all patients. Overall, differences in AUROC across models were modest, whereas XGBoost showed slightly higher AUPRC, suggesting improved identification of higher-risk patients. Pairwise DeLong comparisons using pooled out-of-fold predictions showed no statistically significant AUROC differences between models. AUROC, area under the receiver operating characteristic curve; AUPRC, area under the precision–recall curve; EA, emergence agitation.

**Table 3:** Classification performance of machine learning models at the optimal Youden threshold based on out-of-fold predictions.

Metric	Logistic	XGBoost	Random Forest	CatBoost
Optimal threshold	0.631	0.475	0.553	0.615
Sensitivity	0.727	0.796	0.735	0.680
Specificity	0.805	0.728	0.774	0.796
PPV	0.806	0.766	0.784	0.788
NPV	0.725	0.761	0.723	0.690
Accuracy	0.764	0.764	0.753	0.735
F1-score	0.765	0.780	0.759	0.730

Performance metrics were calculated using pooled out-of-fold predicted probabilities. The optimal classification threshold (probability cutoff) for each model was determined using the Youden index (maximum sensitivity + specificity − 1). All metrics were derived at this threshold. PPV, positive predictive value; NPV, negative predictive value.

## Data Availability

The data presented in this study are available on request from the corresponding author under ethical and legal restrictions related to patient confidentiality.

## Supplementary Materials

The following supporting information can be downloaded at https://www.mdpi.com/article/10.3390/medicina62061189/s1, Table S1: Hyperparameter search space for each machine learning model; Table S2: Global feature importance comparison across machine learning models (aggregated mean absolute SHAP values); Figure S1: Receiver operating characteristic (ROC) and precision–recall (PR) curves for logistic regression based on out-of-fold predictions; Figure S2: Receiver operating characteristic (ROC) and precision–recall (PR) curves for random forest based on out-of-fold predictions; Figure S3: Receiver operating characteristic (ROC) and precision–recall (PR) curves for CatBoost based on out-of-fold predictions.

## Abbreviations

The following abbreviations are used in this manuscript:

EA	emergence agitation
PACU	post-anesthesia care unit
AUROC	area under the receiver operating characteristic curve
AUPRC	area under the precision–recall curve
SHAP	Shapley additive explanations
LMA	laryngeal mask airway
