# Supplementary material for: Explainable Machine Learning Analysis of Perioperative Factors Associated with Clinically Significant Emergence Agitation After Pediatric Ophthalmic Surgery
Source: Medicina (Kaunas). 2026 Jun 19;62(6):1189. doi: 10.3390/medicina62061189 (PMC13303714; doi:10.3390/medicina62061189)
Supplement: Supplementary file 1 [file medicina-62-01189-s001.zip › medicina-4330262-supplementary.pdf]

Supplementary Materials

S1. Hyperparameter Optimization

Hyperparameter tuning was performed using stratified group 5-fold cross-validation at the patient level to prevent data leakage from repeated procedures in the same individual. All hyperparameter searches were conducted strictly within the training folds, and the validation fold was not used during model tuning.

For logistic regression, grid search was conducted over the inverse regularization strength (C) using an L2 penalty. For tree-based models (Random Forest, XGBoost, and CatBoost), randomized search was performed across predefined hyperparameter ranges.

The optimal hyperparameters were selected based on the highest mean area under the receiver operating characteristic curve (AUROC) across cross-validation folds within the training data. The predefined search space for each model is summarized in Supplementary Table S1.

S2. Supplementary Tables

Supplementary Table S1. Hyperparameter search space for each machine learning model.

Model	Hyperparameter	Search range
Logistic regression	C	0.01–10
	Penalty	L2
Random Forest	n_estimators	300–1000
	max_depth	3–10
	min_samples_split	2–10
	min_samples_leaf	1–5
XGBoost	n_estimators	200–800
	max_depth	3–6
	learning_rate	0.01–0.1
	subsample	0.7–1.0
	colsample_bytree	0.7–1.0
	reg_lambda	0–5
CatBoost	iterations	300–800
	depth	4–8
	learning_rate	0.01–0.1
	l2_leaf_reg	1–10

Hyperparameters were optimized exclusively within the training folds using stratified group cross-validation at the patient level. Model selection was based on the highest mean AUROC across folds.

Supplementary Table S2. Global feature importance comparison across machine learning models (aggregated mean absolute SHAP values).

Feature	Logistic	Random Forest	XGBoost	CatBoost
Airway management	1.145	0.175	0.897	1.036
Rocuronium dose	0.185	0.058	0.490	0.590
Induction regimen	0.493	0.086	0.413	0.566
Intraoperative fentanyl dose	0.263	0.042	0.340	0.477
Anesthesia time	0.288	0.027	0.297	0.338
Operation time	0.421	0.032	0.290	0.338
BMI	0.052	0.032	0.262	0.365
Sex	0.130	0.022	0.128	0.191
Age	0.067	0.017	0.072	0.163
Recent URI	0.013	0.003	0.002	0.014
ASA physical status	0.010	0.002	0.000	0.010

SHAP values represent the mean absolute contribution of each feature to model predictions across all patients. Higher values indicate greater global importance. ASA, American Society of Anesthesiologists; BMI, body mass index; URI, upper respiratory infection.

S3. Supplementary Figures Legend

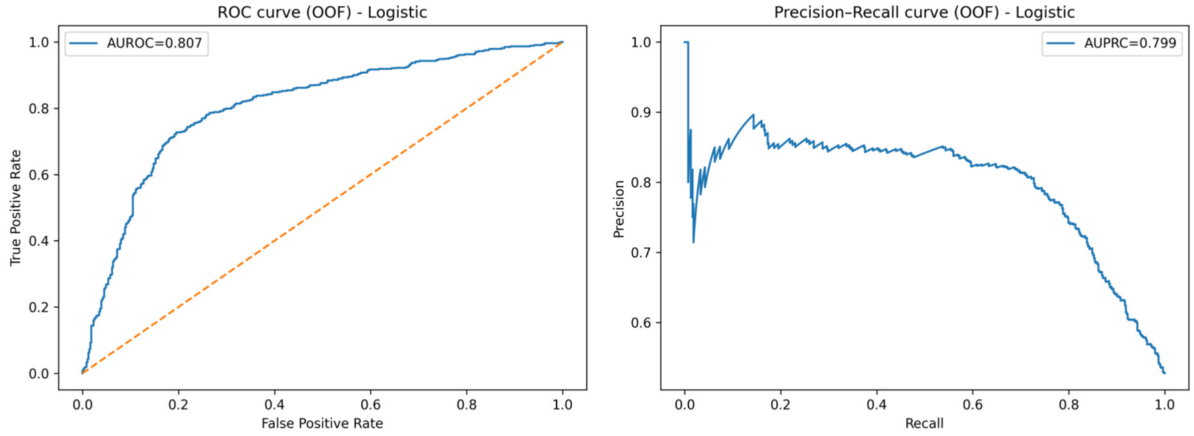

Supplementary Figure S1. Receiver operating characteristic (ROC) and precision–recall (PR) curves for logistic regression based on out-of-fold predictions.

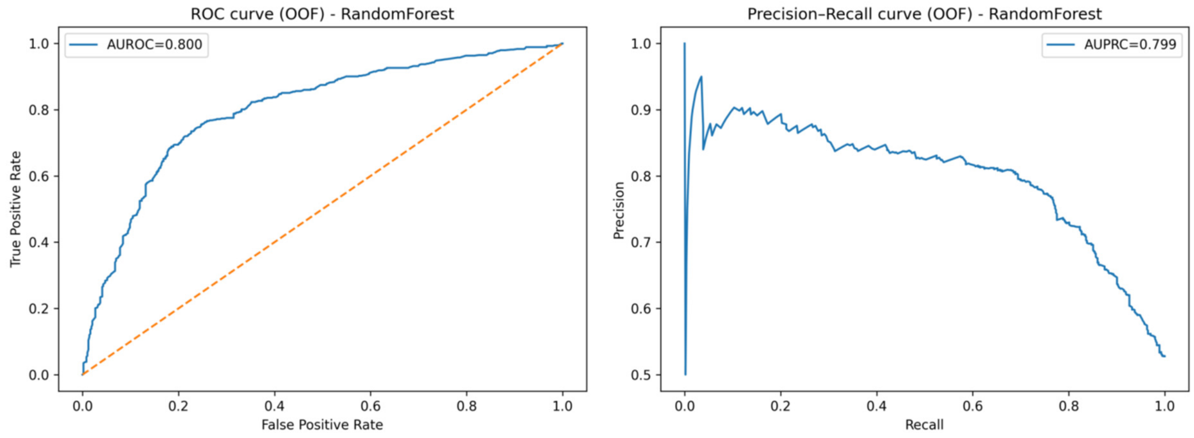

Supplementary Figure S2. Receiver operating characteristic (ROC) and precision–recall (PR) curves for random forest based on out-of-fold predictions.

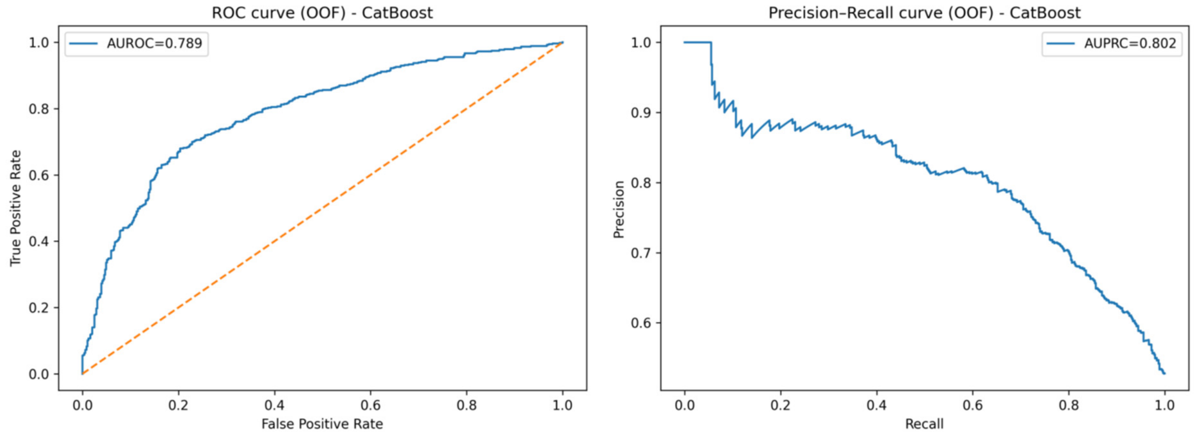

Supplementary Figure S3. Receiver operating characteristic (ROC) and precision–recall (PR) curves for CatBoost based on out-of-fold predictions.

All curves were generated using pooled out-of-fold predicted probabilities obtained from stratified group 5-fold cross-validation at the patient level.
