# Supplementary figures and images for: Explainable Machine Learning Analysis of Perioperative Factors Associated with Clinically Significant Emergence Agitation After Pediatric Ophthalmic Surgery
Source: Medicina (Kaunas). 2026 Jun 19;62(6):1189. doi: 10.3390/medicina62061189 (PMC13303714; doi:10.3390/medicina62061189)

ROC curve (OOF) - Logistic

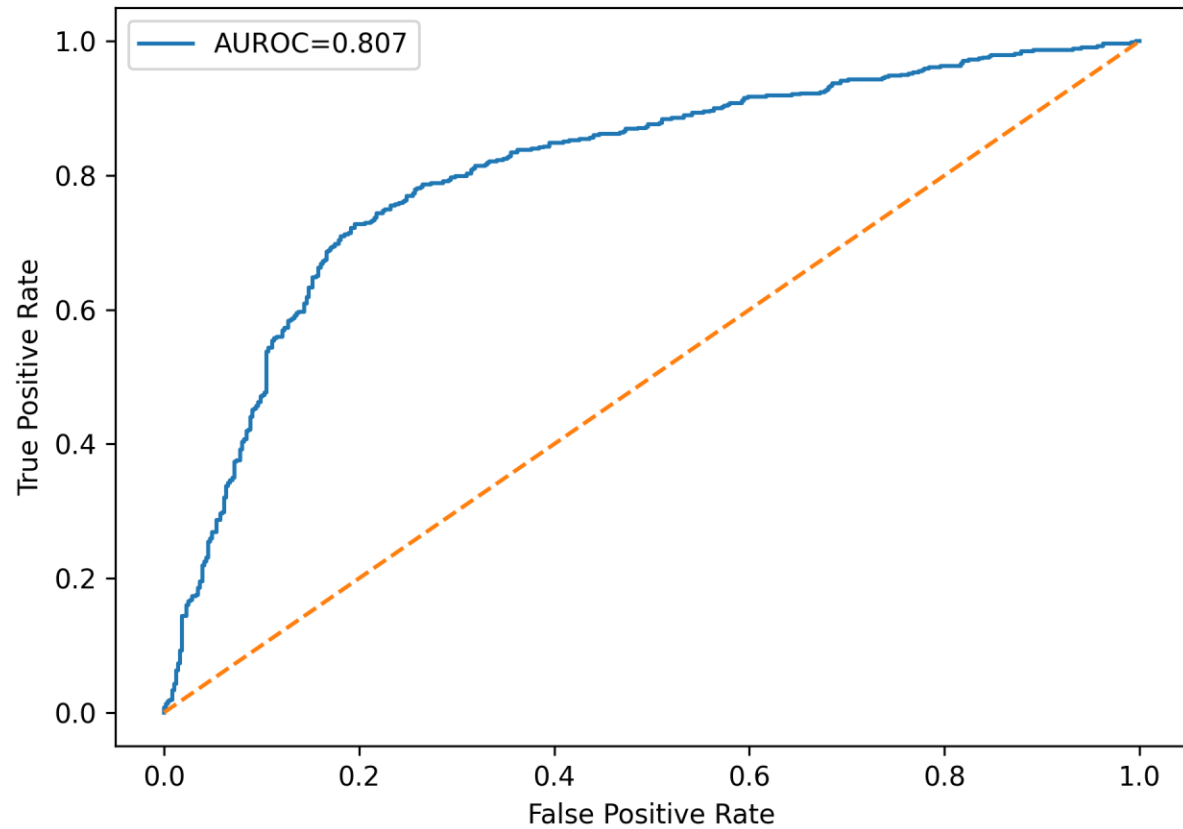

Precision-Recall curve (OOF) - Logistic

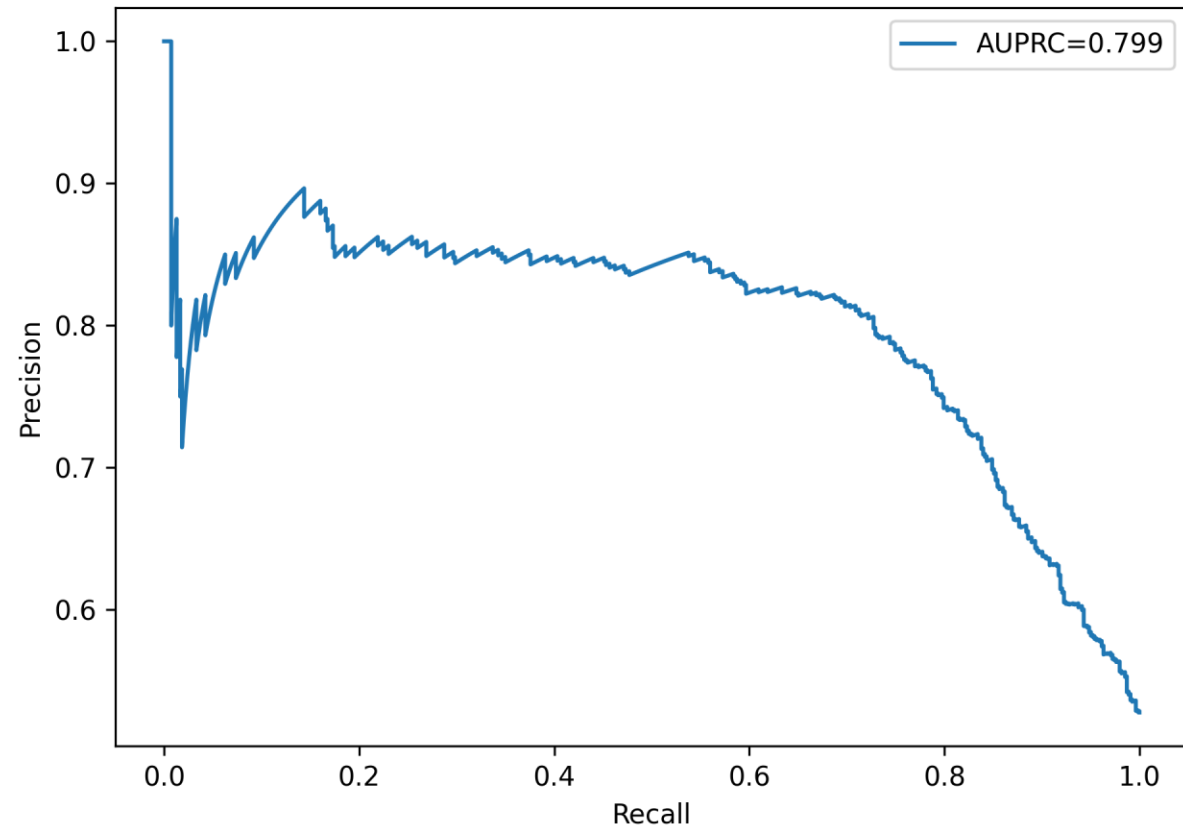

Supplement: Supplementary file 1 [file medicina-62-01189-s001.zip › Supplementary Figure S1.pdf]

ROC curve (OOF) - RandomForest

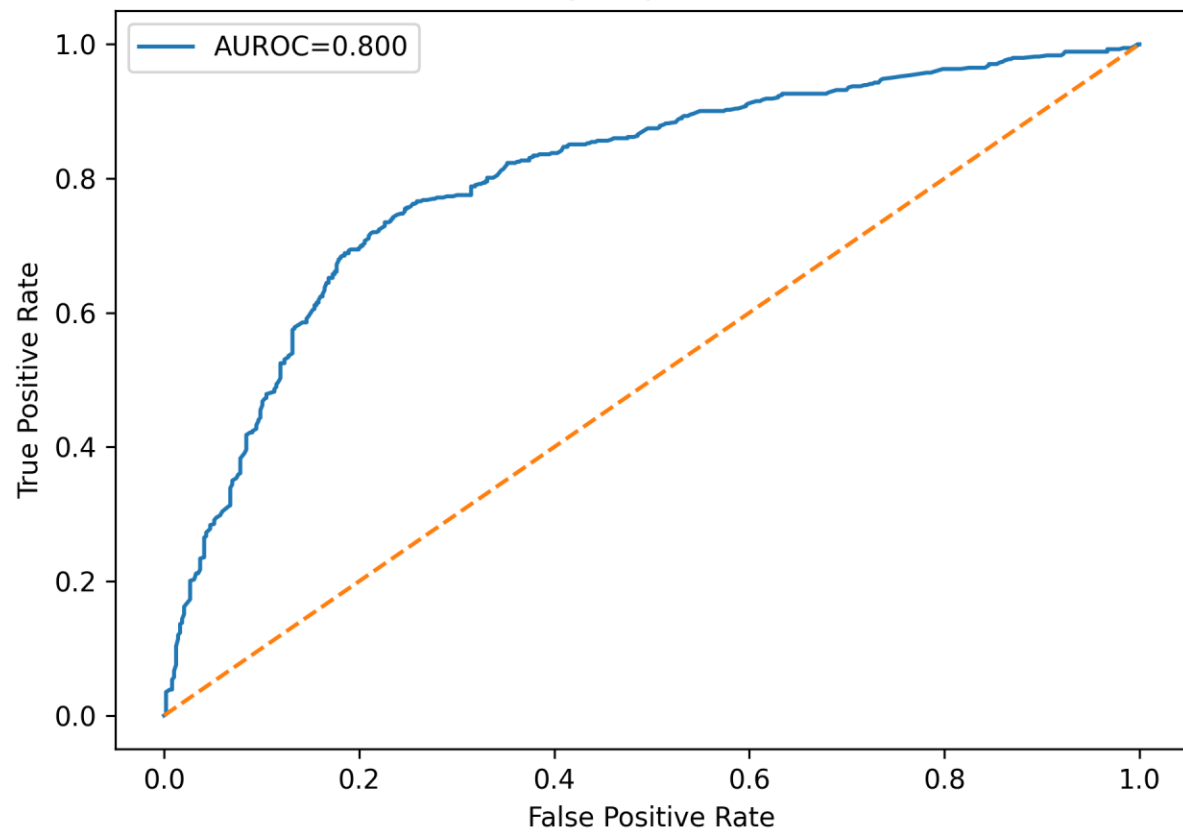

Precision-Recall curve (OOF) - RandomForest

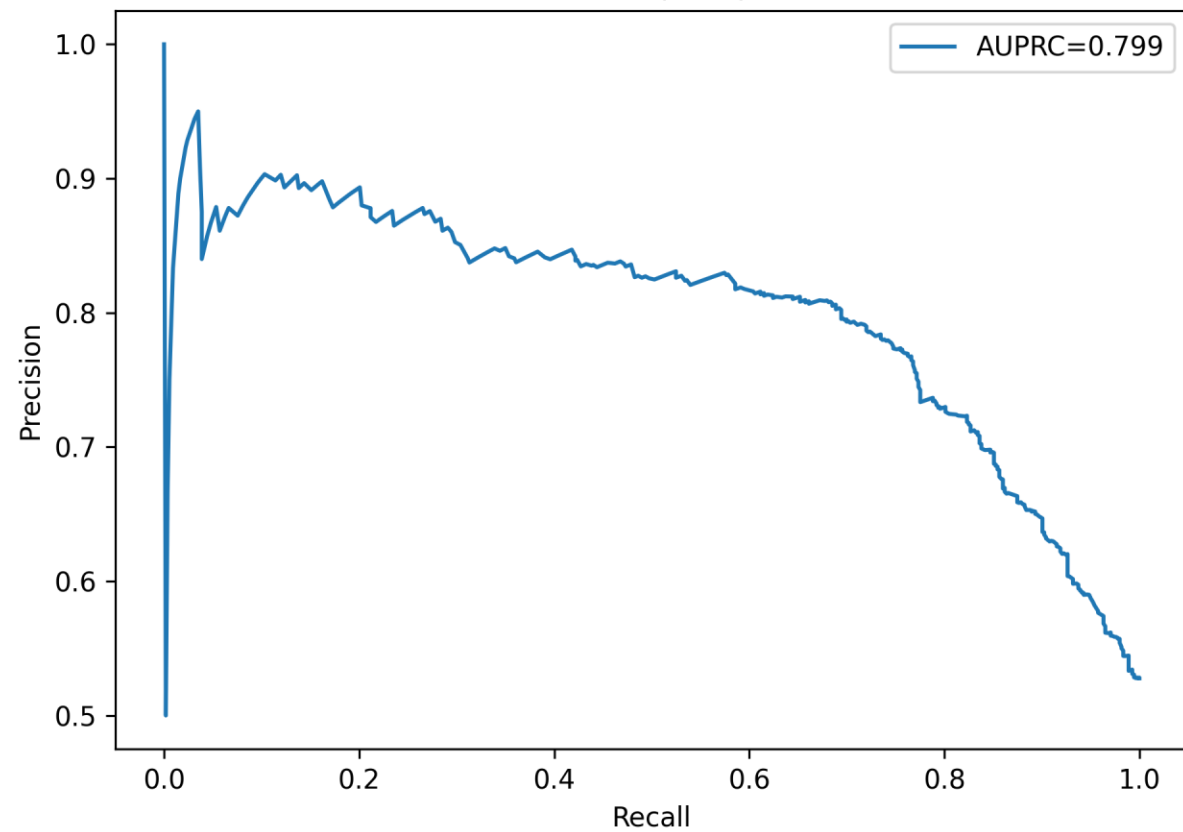

Supplement: Supplementary file 1 [file medicina-62-01189-s001.zip › Supplementary Figure S2.pdf]

ROC curve (OOF) - CatBoost

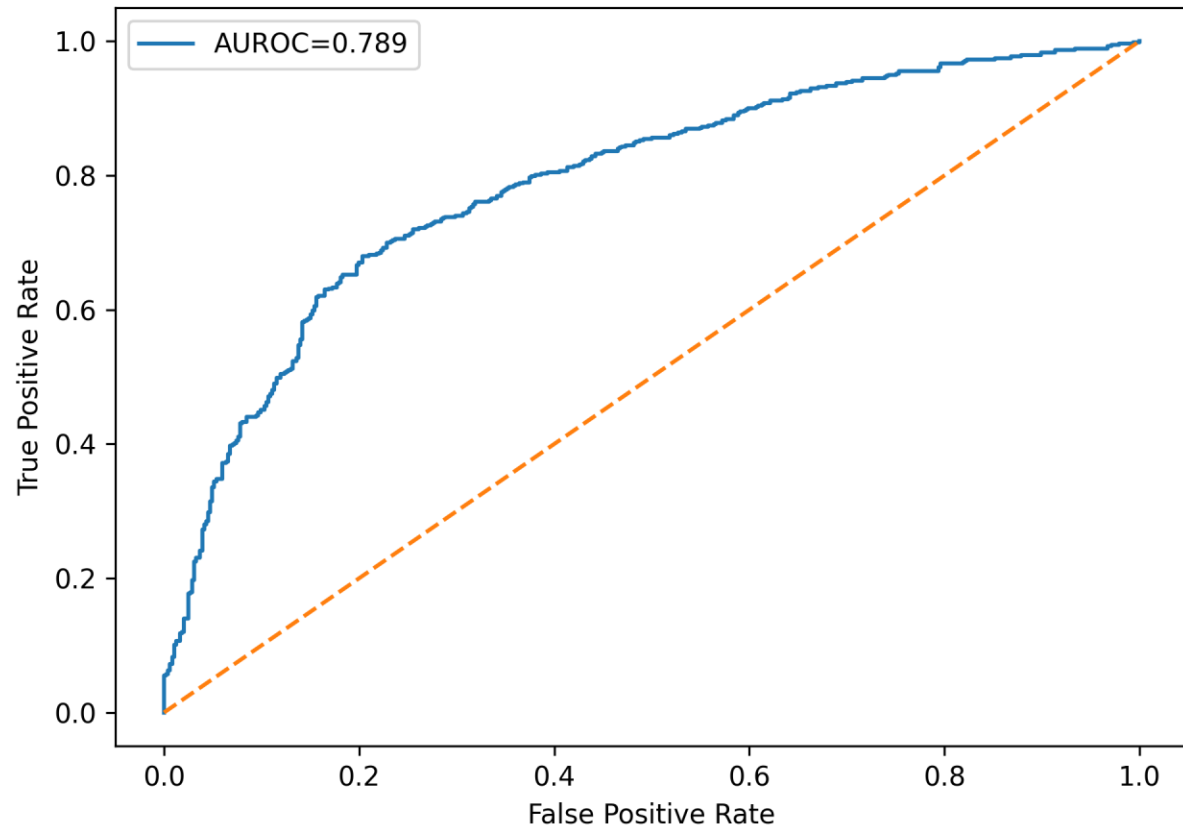

Precision-Recall curve (OOF) - CatBoost

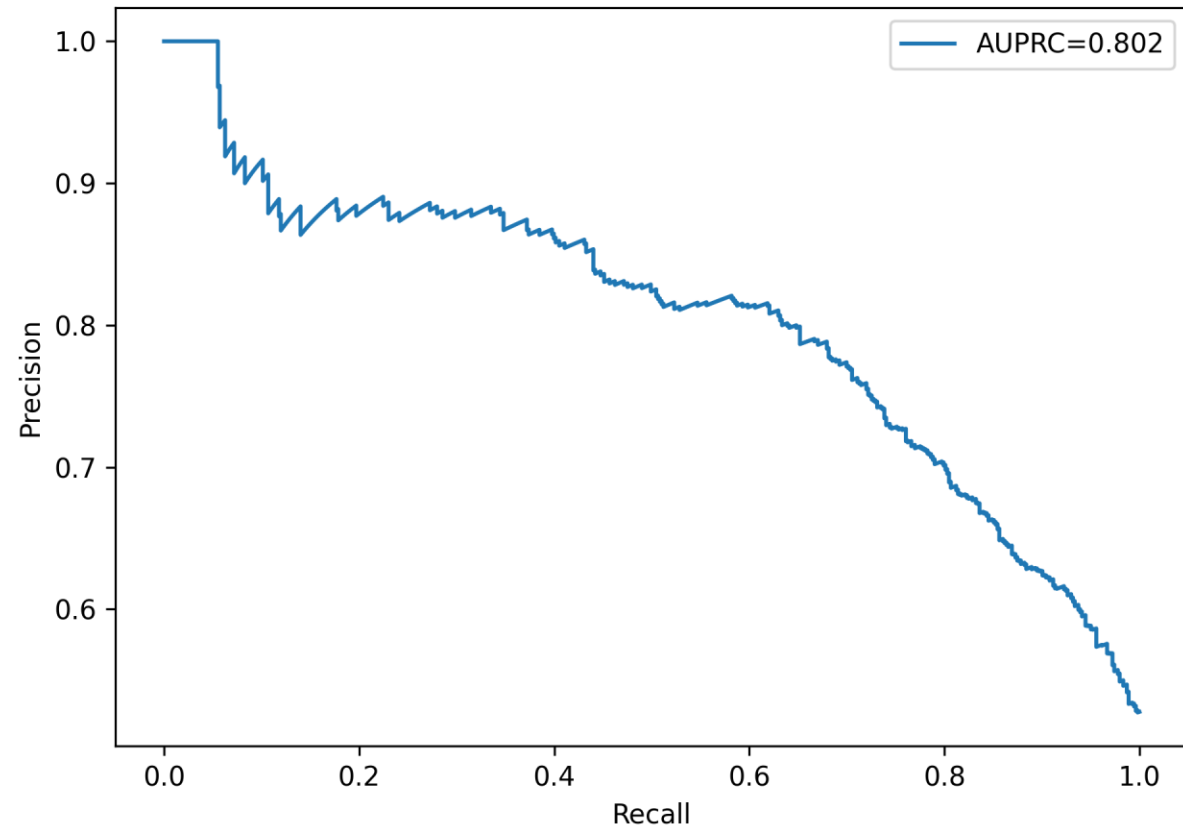

Supplement: Supplementary file 1 [file medicina-62-01189-s001.zip › Supplementary Figure S3.pdf]
